# Can Massive Religious Festival Celebrations Encourage a Faster Spread of a Pandemic? The Case of COVID-19 in Israel

**DOI:** 10.1007/s10943-024-02153-x

**Published:** 2024-11-01

**Authors:** Mario Arturo Ruiz Estrada, Inna Levy

**Affiliations:** 1 University of Economics and Human Sciences (UEHS), Okopowa 59, 01-043 Warsaw, Poland; 2 Department of Criminology, Ariel University, Israel and Department of Interdisciplinary Studies, Zefat Academic College, Israel, 13206 Zefat, Israel

**Keywords:** Israel, COVID-19, Religion, Pandemics, Vaccination, A1

## Abstract

This paper attempts to evaluate how massive religious festival celebrations can encourage the faster spread of any pandemic according to our problem statement, such as the case of COVID-19. For example, we evaluate Israel’s three major religions, namely  Judaism, Christianity, and the Islamic festival  celebrations, respectively. Firstly, we have the traditional Jewish festivities such as Hanukkah, Yom Kippur, Sukkot, and Rosh Hashanah. In the Christian’s traditional festivities celebrations, we identified Christmas, Easter Day, and All Saints Day. Finally, the Muslim festivities of Muharram and the Birthday of the Holy Prophet Mohamad. The purpose of  this study was to evaluate if these nine massive religious festival  celebrations are the main reasons for the large spread of COVID-19 in Israel directly or indirectly. In fact, we propose a new methodology to evaluate the impact of any massive religious festival celebration and the fast spread of any pandemic everywhere and anytime. The new indicator is entitled “The National Spread Levels of Infectious Diseases Risk from Massive Religious Festivities Index” (National-SLIDRMRF-Index). Finally, the major finding in this research is that any massive religious events can generate an exponential number of COVID-19 cases constantly. Therefore, this research concluded that we urgently need a standardized index to monitor  and control the expansion of any pandemic such as COVID-19 among different religious groups in the same country. At the same time, we give different policy recommendations to the Israeli government to constantly keep major controls and measures of different religious events in Jerusalem.

## Introduction

The COVID-19 pandemic originated in Wuhan, China, in December 2019 (Ren et al., 2020). On January 27, 2020, the Israeli Minister of Health signed an order which required immediate notification of authorities in cases of COVID-19, and about a month later, on February 21, 2020, the first COVID-19 case in Israel was confirmed (Last, 2020). On the same day, the Israeli government established a 14-day home quarantine rule for people who visited South Korea and Japan. By March 9, the 14-day home quarantine was extended for all people entering Israel (Jaffe-Hoffman, 2020).

As time went by, more and more individuals in Israel were diagnosed with COVID-19 and sent to quarantine, but there were no substantial restrictions on social interactions and group and mass gatherings. On March 2, Israeli citizens participated in Israeli legislative elections, and between the 10th and 11th of March, 2020, Israeli Jews celebrated a Jewish holiday of Purim. These two events lead to more confirmed cases of COVID-19 (Coronavirus Resource Center, 2020).

Following March 10, the Israeli government began gradually limiting gatherings and increasing restrictions. Thus, if on March 10, gatherings of more than 2,000 were banned, then by March 15, all gatherings of more than ten people were banned. Additionally, Israeli government granted Israeli security service a permission to perform mass surveillance to insure that citizens comply with the quarantine order (Gross, 2020).

Furthermore, on March 13, all educational institutions, day centers, after school programs were closed (Stein-Zamir et al., 2020). By March 19, due to the daily growing number of confirmed cases, the government ordered to close all non-essential businesses, the social distances increased, and citizens movements outside their homes were significantly limited (Last, 2020). Thus, the first lockdown lasted from March 16 to April 19, 2020 (Birenbaum-Carmeli & Chassida, 2020).

This time period included Jewish Passover, during which additional restrictions were imposed. There was also Ramadan-related partial lockdown. The second lockdown was from September 13, 2020, to October 18, 2020. This lockdown took place during such major Jewish holydays as Rosh Hashana (Jewish New Year), Yom Kipur, and Sukkot. During these lockdowns, school and non-essential business and organizations were closed, indoor praying and gatherings of more than 10 people were banned, public transport limited, and citizens were restricted regarding their movements outside of their homes. On holiday evenings, cities were closed to prevent families from celebrating together (Griver, 2020; The Times of Israel, 2020).

Moreover, following US FDA emergency approval of Pfizer-BioNTech COVID-19 vaccine and Israeli’s Ministry of Health (MOH) authorization, on December 20, 2020, Israeli government launched COVID-19 vaccination campaign. By the end of 2020, Israel administered more vaccine doses than most of the countries (except China, USA, and UK) and at the highest rate (Rosen et al., 2021). At first, the vaccine was administrated to people ages 60 and older and by now 90% of this group has been vaccinated. Consequently, data indicate a 41% decrease in confirmed cases of COVID-19 in this age group (Mallapaty, 2021). According to MOH, incidence rates in fully vaccinated population significantly decreased compared to the unvaccinated population, including a decline in hospitalization due to COVID-19 (Tercatin, 2021).

However, the research objective of this paper is the construction and evaluation of different religions celebrations under the application of a single index to monitoring COVID-19 spread cases constantly. According to this research, we have three main objectives:

- To monitoring large religious activities in different spatiotemporal patterns constantly.
- Major controls and regulations in any religious activity systematically.
- The mapping of critical areas according to the religious groups density located in different parts of Israel to have a better control of the COVID-19-infected cases.

All these main objectives are related to our research problem statement that is to evaluate how massive religious festivities celebrations can encourage the faster spread of any pandemic.

The idea to write this paper is originated by the massive religions activities as the main reason of the fast COVID-19 spread cases in Israel. According to this research paper, the cyclical movements in the expansion or contraction of the number of COVID-19-infected cases have its origins from the massive agglomerations in different religions celebrations according to different dates.

Additionally, the same paper introduces an original model that is presenting a serial of equations, limits, and conditions that facilitate the explanation of exogenous and endogenous variables in our analysis. In fact, our model applies first derivatives to represent the maximum and minimum of COVID-19-infected cases to analyze the cyclical behavior of it annually, at the same time, the application of benefit/cost to analyze the relationship between COVID-19-infected cases and the control of people mobility in religious celebrations.

Finally, the great contribution of this research paper is the introduction of a new collusion model to support the control of massive religious celebrations and punishment through the establishment of strict rules and measurements, respectively. Therefore, we find that major part of models about massive religious celebrations events in the spread of COVID-19-infected cases is based on the analysis of benefit/cost (opportunity/punishment), comparative historical data (absolute and relative values), correlations and forecasting (Quadri, 2020) (Tan et al., 2022).

Another important research paper to be mentioned in this research is from Lee et al. (2021). All these authors mentioned before extend the analysis of the impact of large religious celebrations events impact in the pandemic times such as the case of COVID-19 by using different research approaches such as qualitative (legal) and quantitative (economics), respectively.

In our case, we like to propose a new model to monitoring the impact of large religious celebrations in the spread of COVID-19. The central objective of our paper is to set forth a new model—”The National Spread Levels of Infectious Diseases Risk from Massive Religious Festivities Index (National-SLIDRMRF-Index)”—to evaluate the impact of large religious celebration events to avoid the fast spread of COVID-19 infections cases systematically.

The main methodology we are using is econographicology (Ruiz Estrada, 2017) to reproduce and evaluate the fast spread of COVID-19-infected cases originated from massive religious gatherings cyclically from a multi-dimensional point of view. At the same time, the data we are using are from first sources by Coronavirus Resource Center (2020), *COVID-19 Dashboard by the Center for Systems Science and Engineering (CSSE) at Johns Hopkins University (JHU) (2021),* and WHO (2021), respectively,

## An Introduction to the National Spread Levels of Infectious Diseases Risk from Massive Religious Festivities Index (National-SLIDRMRF-Index)

The national spread levels of infectious diseases risk from massive religious festivities index (National-SLIDRMRF-Index) offer us an alternative indicator to analyze the religious massive festivities impact on the fast spread of COVID-19 from a multi-dimensional perspective at the national level. In fact, the National-SLIDRMRF-Index offers policy makers (Ruiz Estrada, 2011), religious leaders, academics, and governments an alternative model to control massive pandemics on large religious events in the same country such as Israel. The National-SLIDRMRF-Index has three vectors interacting in real time under the application of millions of dynamic growth rates simultaneously (See Expression 1.1, 1.2, and 1.3).

Vector 1: Jewish population infected by COVID-19 daily

1.1 $$\sun{\text{V}}_{{1}} = \, (\sun \Delta \alpha_{{{1}:0}} ,\sun \Delta \alpha_{{{1}:{1}}} ,\sun \Delta \alpha_{{{1}:{2}}} , \ldots ,\sun \Delta \alpha_{{{1}:\infty }} \ldots )$$

Vector 2: Christians Population infected by COVID-19 daily

1.2 $$\sun{\text{V}}_{{2}} = \, f \, (\sun \Delta \alpha_{{{2}:0}} ,\sun \Delta \alpha_{{{2}:{1}}} ,\sun \Delta \alpha_{{{2}:{2}}} , \ldots ,\sun \Delta \alpha_{{{2}:\infty }} \ldots )$$

Vector 3: Muslims Population infected by COVID-19 daily

1.3 $$\sun{\text{V}}_{{3}} = \, f \, (\sun \Delta \alpha_{{{3}:0}} ,\sun \Delta \alpha_{{{3}:{1}}} ,\sun \Delta \alpha_{{{3}:{2}}} , \ldots ,\sun \Delta \alpha_{{{3}:\infty }} \ldots )$$

☼ = Real Time Vi = Vector ∆ = Dynamic Growth Rate Daily.

Each dynamic growth rate daily (☼∆α_i:j_) in each dynamic vectors requires the application of the expression 1.4.

1.4 $$\sun \Delta \alpha_{{{\text{i}}:{\text{j}}}} = \sun \Delta \alpha_{{{\text{i}}:{\text{j }} < {\text{t}} + {1} > }} - \sun \Delta \alpha_{{{\text{i}}:{\text{j}} < {\text{t}}0 > }} {\text{x 1}}00\%$$

☼∆α_i:j<t0>_

Note: i = {1, 2, 3}, j = {0, 1, 2,…∞}, < t + 1 >  = the number of infected cases from COVID-19 today, < t_o_ >  = the number of infected cases from COVID-19 yesterday.

Therefore, the final mathematic structure is to build the National-LIDRMRF-Index under the application of expression (2)

2 $${\text{National}} - {\text{SLIDRMRF}} - {\text{Index}} \equiv \sun{\text{V}}_{{1}} \mathop{\lrcorner\llcorner}\limits_{\urcorner\ulcorner} \sun{\text{V}}_{{2}} \mathop{\lrcorner\llcorner}\limits_{\urcorner\ulcorner} \sun{\text{V}}_{{3}}$$

Note: ╬ = linkage of quadrants.

The application of the National-SLIDRMRF-Index requests to follow three basic steps. The first step is to build each dynamic growth rate daily (see Expression 1.4). The second step is constructing each dynamic vector for each religion in our study: Judaism, Christianity, and Islam (see Expression 1.1, 1.2, and 1.3). The third step requires comparing three dynamic vectors graphically under three surfaces’ uses to observe the impact of COVID-19 in each religious group easily. This helps us evaluate the final impact of massive religious festivities celebrations in the spread of COVID-19 in Israel.

**Fig. 1:**
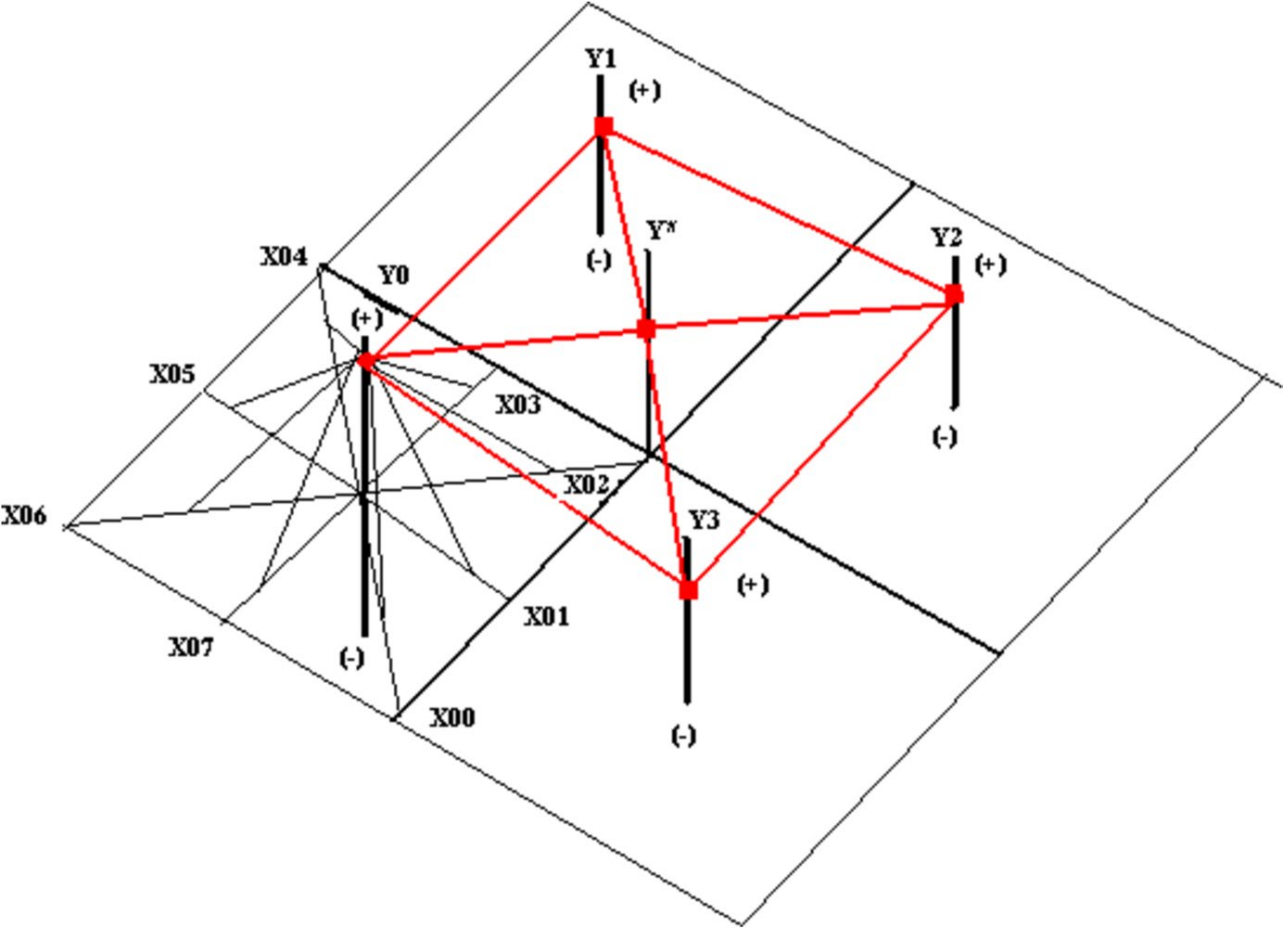
Sub-RMMPC-function. *Source* (Ruiz Estrada, 2017)

The main objective of the National-SLIDRMRF-Index is to monitor the effects of the COVID-19 on those three religious’ groups in Israel. The first step is to try to find the final national religious events in three dynamic vectors, based on measuring each dynamic vector represented by the Jewish, Christians, and Muslims. Finally, each religious group’s dynamic vectors require joining all dynamic sub-vectors in the common axis shared on the same surface (see Expression 2 and Fig. 1).

**Fig. 2:**
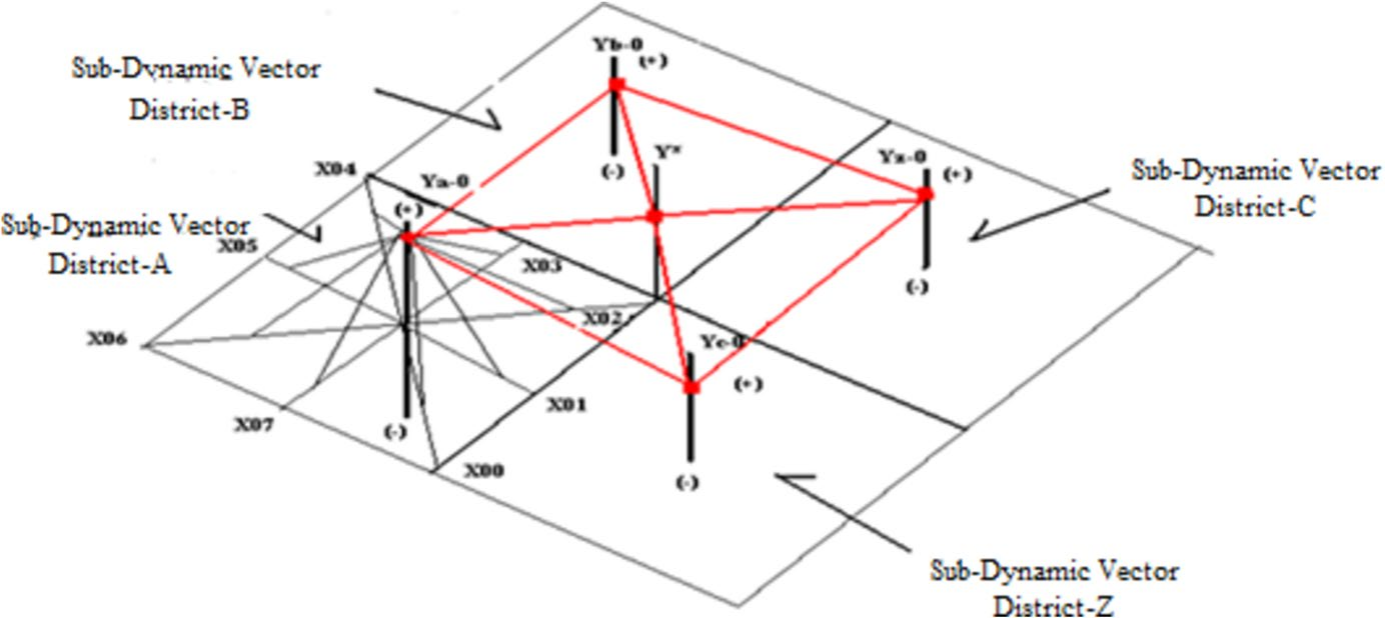
Sub-dynamic vectors from district-A to district-Z. *Source* (Ruiz Estrada, 2017)

The second step is to compare the three dynamic vectors graphically. The third step is to assess which district in Israel is more affected by the COVID-19. The three dynamic vectors assessment provides information about the reorganization of future religious festivities celebrations by district (see Expression 3.1, 3.2, 3.2, and Fig. 2).

The sub-dynamic vector for Jewish population is represented by 3.1.

3.1 $$\begin{gathered} {\text{Sub - Dynamic Vector 0{-\!\!-}District }}^{\prime \prime } A^{\prime \prime } : \hfill \\ \sun SV - A_{0:1:j} = \, f \, (\sun \Delta \alpha A_{0:1:0} ,\sun \Delta \alpha A_{0:1:1} ,\sun \Delta \alpha A_{0:1:2} , \ldots ,\sun \Delta \alpha A_{0:1:\infty } \ldots ) \hfill \\ {\text{Sub - Dynamic Vector 1{-\!\!-}District }}^{\prime \prime } B^{\prime \prime } : \hfill \\ \sun SV - B_{1:1:j} = \, f \, (\sun \Delta \alpha B_{1:1:0} ,\sun \Delta \alpha B_{1:1:1} ,\sun \Delta \alpha B_{1:2:2} , \ldots ,\sun \Delta \alpha B_{1:1:\infty } \ldots ) \hfill \\ {\text{Sub - Dynamic Vector 2{-\!\!-}District }}^{\prime \prime } C^{\prime \prime } : \hfill \\ \sun SV - C_{2:1:j} = \, f \, (\sun \Delta \alpha C_{2:1:0} ,\sun \Delta \alpha C_{2:1:1} ,\sun \Delta \alpha C_{2:1:2} , \ldots ,\sun \Delta \alpha C_{2:1:\infty } \ldots ) \hfill \\ {\text{Sub - Dynamic Vector n{-\!\!-}District }}^{\prime \prime } Z^{\prime \prime } : \hfill \\ \sun SV - Z_{n:1:n} = \, f \, (\sun \Delta \alpha Z_{n:1:0} ,\sun \Delta \alpha Z_{n:1:1} ,\sun \Delta X\alpha_{n:1:2} , \ldots ,\sun \Delta \alpha Z_{n:1:\infty } \ldots ) \hfill \\ \end{gathered}$$

The Sub-RMMPC-Functions for the Christian population (see Expression 3.2).

3.2 $$\begin{gathered} {\text{Sub - Dynamic Vector 0{-\!\!-}District }}^{\prime \prime } A^{\prime \prime } : \hfill \\ \sun SV - A_{0:2:j} = \, f \, (\sun \Delta \alpha A_{0:2:0} ,\sun \Delta \alpha A_{0:2:1} ,\sun \Delta \alpha A_{0:2:2} , \ldots ,\sun \Delta \alpha A_{0:2:\infty } \ldots ) \hfill \\ {\text{Sub - Dynamic Vector 1{-\!\!-}District }}^{\prime \prime } B^{\prime \prime } : \hfill \\ \sun SV - B_{1:2:j} = \, f \, (\sun \Delta \alpha B_{1:2:0} ,\sun \Delta \alpha B_{1:2:1} ,\sun \Delta \alpha B_{1:2:2} , \ldots ,\sun \Delta \alpha B_{1:2:\infty } \ldots ) \hfill \\ {\text{Sub - Dynamic Vector 2{-\!\!-}District }}^{\prime \prime } C^{\prime \prime } : \hfill \\ \sun SV - C_{2:2:j} = \, f \, (\sun \Delta \alpha C_{2:2:0} ,\sun \Delta \alpha C_{2:2:1} ,\sun \Delta \alpha C_{2:2:2} , \ldots ,\sun \Delta \alpha C_{2:2:\infty } \ldots ) \hfill \\ {\text{Sub - Dynamic Vector n{-\!\!-}District }}^{\prime \prime } Z^{\prime \prime } : \hfill \\ \sun SV - Z_{n:2:n} = \, f \, (\sun \Delta \alpha Z_{n:2:0} ,\sun \Delta \alpha Z_{n:2:1} ,\sun \Delta X\alpha_{n:2:2} , \ldots ,\sun \Delta \alpha Z_{n:2:\infty } \ldots ) \hfill \\ \end{gathered}$$

The Sub-RMMPC-Function for the Muslim population according to Expression 3.3.

3.3 $$\begin{gathered} {\text{Sub - Dynamic Vector 0{-\!\!-}District }}^{\prime \prime } A^{\prime \prime } : \hfill \\ \sun SV - A_{0:3:j} = \, f \, (\sun \Delta \alpha A_{0:3:0} ,\sun \Delta \alpha A_{0:3:1} ,\sun \Delta \alpha A_{0:3:2} , \ldots ,\sun \Delta \alpha A_{0:3:\infty } \ldots ) \hfill \\ {\text{Sub - Dynamic Vector 1{-\!\!-}District }}^{\prime \prime } B^{\prime \prime } : \hfill \\ \sun SV - B_{1:3:j} = \, f \, (\sun \Delta \alpha B_{1:3:0} ,\sun \Delta \alpha B_{1:3:1} ,\sun \Delta \alpha B_{1:3:2} , \ldots ,\sun \Delta \alpha B_{1:3:\infty } \ldots ) \hfill \\ {\text{Sub - Dynamic Vector 2{-}District }}^{\prime \prime } C^{\prime \prime } : \hfill \\ \sun SV - C_{2:3:j} = \, f \, (\sun \Delta \alpha C_{2:3:0} ,\sun \Delta \alpha C_{2:3:1} ,\sun \Delta \alpha C_{2:3:2} , \ldots ,\sun \Delta \alpha C_{2:3:\infty } \ldots ) \hfill \\ {\text{Sub - Dynamic Vector n{-\!\!-}District }}^{\prime \prime } Z^{\prime \prime } : \hfill \\ \sun SV - Z_{n:3:\infty } = \, f \, (\sun \Delta \alpha Z_{0:3:0} ,\sun \Delta \alpha Z_{0:3:1} ,\sun \Delta X\alpha_{0:3:2} , \ldots ,\sun \Delta \alpha Z_{0:3:\infty } \ldots ) \hfill \\ \end{gathered}$$

Suppose a country in a high number of COVID-19 cases from the sub-dynamic vector zero, followed by (☼SV-A_(i = 0,1,2,…,∞):(j = 1,2,3):(k = 0,1,2,…,∞)_) ∩ (☼SV-B_(i = 0,1,2,…,∞):(j = 1,2,3):(k = 0,1,2,…,∞)_) ∩ (☼SV-C_(i = 0,1,2,…,∞):(j = 1,2,3):(k= 0,1,2,…,∞)_) ∩ (☼SV-Z_(i = 0,1,2,…,∞):(j = 1,2,3):(k = 0,1,2,…,∞)_). Then this religion group festivities celebrations need to be reorganized according to its sub-dynamic vectors behavior, in our case the Jewish (j = 1), Christians (j = 2), and Muslims (j = 3) population.

**Fig. 3:**
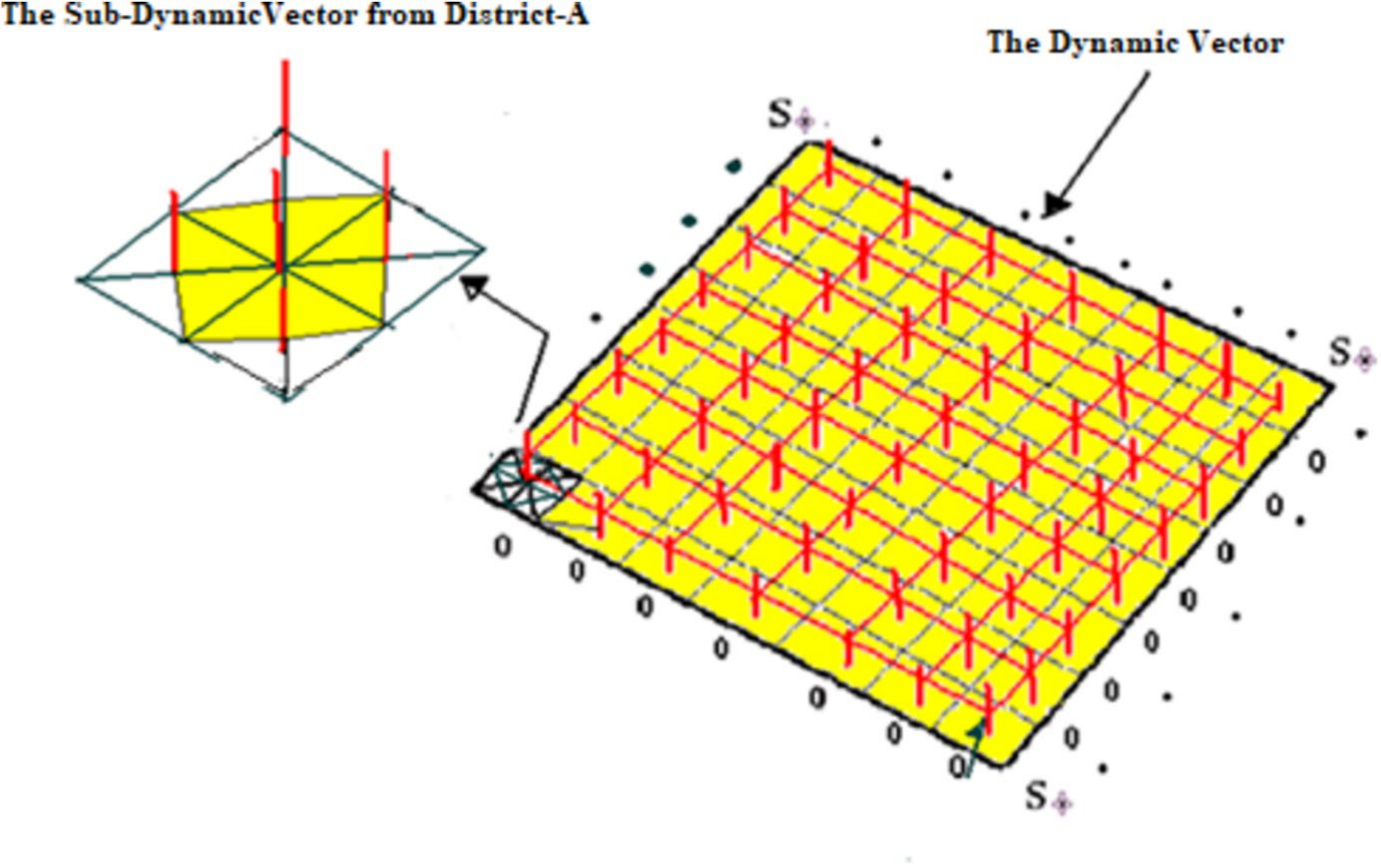
Construction of surfaces under the uses of sub-dynamic vectors and the dynamic vector. *Source* (Ruiz Estrada, 2017)

It is based on joining all sub-dynamic vectors by the main three religions of Israel by using straight lines until we can draw a single surface (see Fig. 3). The three dynamic vectors (1.1, 1.2, 1.3) outcome depends on the location of the surface, which is determined by low COVID-19-infected cases (see Expression 4.1), partial COVID-19-infected cases (see Expression 4.2), irregular COVID-19-infected cases (see Expression 4.3), and high COVID-19-infected cases (see Expression 4.4). There are four possible locations.

4.1 $${\text{National}} - {\text{SLIDRMRF}} - {\text{Index}} \equiv \sun + V_{1} \mathop{\lrcorner\llcorner}\limits_{\urcorner\ulcorner} \sun + V_{2} \mathop{\lrcorner\llcorner}\limits_{\urcorner\ulcorner} \sun + V_{3}$$

{If any dynamic vector (V_i =1,2,3_) ∩ R_+_ then the surface ≡ low COVID-19-infected cases}

4.2 $${\text{National}} - {\text{SLIDRMRF}} - {\text{Index}} \equiv \sun V_{1} = \, 0\mathop{\lrcorner\llcorner}\limits_{\urcorner\ulcorner} \sun V_{2} = \, 0\mathop{\lrcorner\llcorner}\limits_{\urcorner\ulcorner} \sun V_{3} = \, 0$$

{if any dynamic vector (V_i=1,2,3_) ∩ 0 then the surface ≡ partial COVID-19-infected cases}

4.3 $${\text{National}} - {\text{SLIDRMRF}} - {\text{Index}} \equiv \sun + / - V_{1} \mathop{\lrcorner\llcorner}\limits_{\urcorner\ulcorner} \sun + / - V_{2} \mathop{\lrcorner\llcorner}\limits_{\urcorner\ulcorner} \sun + / - V_{3}$$

{if any dynamic vector (V_i=1,2,3_) ∩ R ± then the surface ≡ irregular COVID-19-infected cases}

4.4 $${\text{National}} - {\text{SLIDRMRF}} - {\text{Index}} \equiv \sun - V_{1} \mathop{\lrcorner\llcorner}\limits_{\urcorner\ulcorner} \sun - V_{2} \mathop{\lrcorner\llcorner}\limits_{\urcorner\ulcorner} - V_{3}$$

{if any dynamic vector (V_i=1,2,3_) ∩ R- then the surface ≡ high COVID-19-infected cases}.

## Application of the National Spread Levels of Infectious Diseases Risk from Massive Religious Festivities Index (National-SLIDRMRF-Index) in Israel

**Fig. 4:**
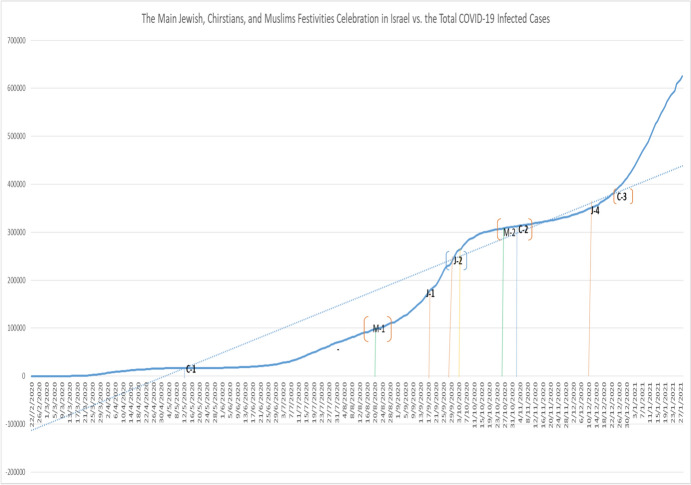
Main Jewish, Christians, and Muslims festivities celebrations in Israel versus the total COVID-19-infected cases. *Source* Centre for Diseases Control and Prevention (2021), Ministry of Health of Israel (2021), and WHO (2021)

We apply the National-SLRDRMRF-Index to Israel between 2020 and 2021. Initially, we try to evaluate Israel’s major religious festivities celebrations. Firstly, we have the traditional Jewish festivities celebrations followed by Yom Kippur “J-1” (27–28 September 2020), Sukkot “J-2” (2–9 October 2020), Rosh Hashanah “J-3” (18–20 September 2020), and Hanukkah “J-4” (10–18 December 2020) (see Fig. 4).

In the Christian’s traditional festivities celebrations, we identified Easter Day “C-1” (12 April 2020), All Saints Day “C-2” (1 November 2020), and Christmas “C-3” (24–25 December 2020). Finally, the Muslim festivities celebrations present the Muharram “M-1” (20–21 August 2020) and the Birthday of Holy Prophet Mohamad “M-2” (29 October 2020). In these graphs, we can observe each religious festivity celebration in Israel and the total number of COVID-19 cases daily in the year 2020 and beginning of the year 2021.

According to preliminary results, the first wave of COVID-19 started with Muharram “M-1” (20–21 August 2020), the cases of COVID-19 starting to increase considerably in all Israel; the second wave started with the Sukkot “J-2” (2–9 October 2020). The third wave of COVID-19 is related to All Saints Day “C-2” (1 November 2020) and the Birthday of Holy Prophet Mohamad “M-2” (29 October 2020). The fourth wave of COVID-19 is related to Hanukkah “J-4” (10–18 December 2020). And finally, the fifth wave of COVID-19 is the celebration of Christmas “C-3” (24–25 December 2020).

**Fig. 5:**
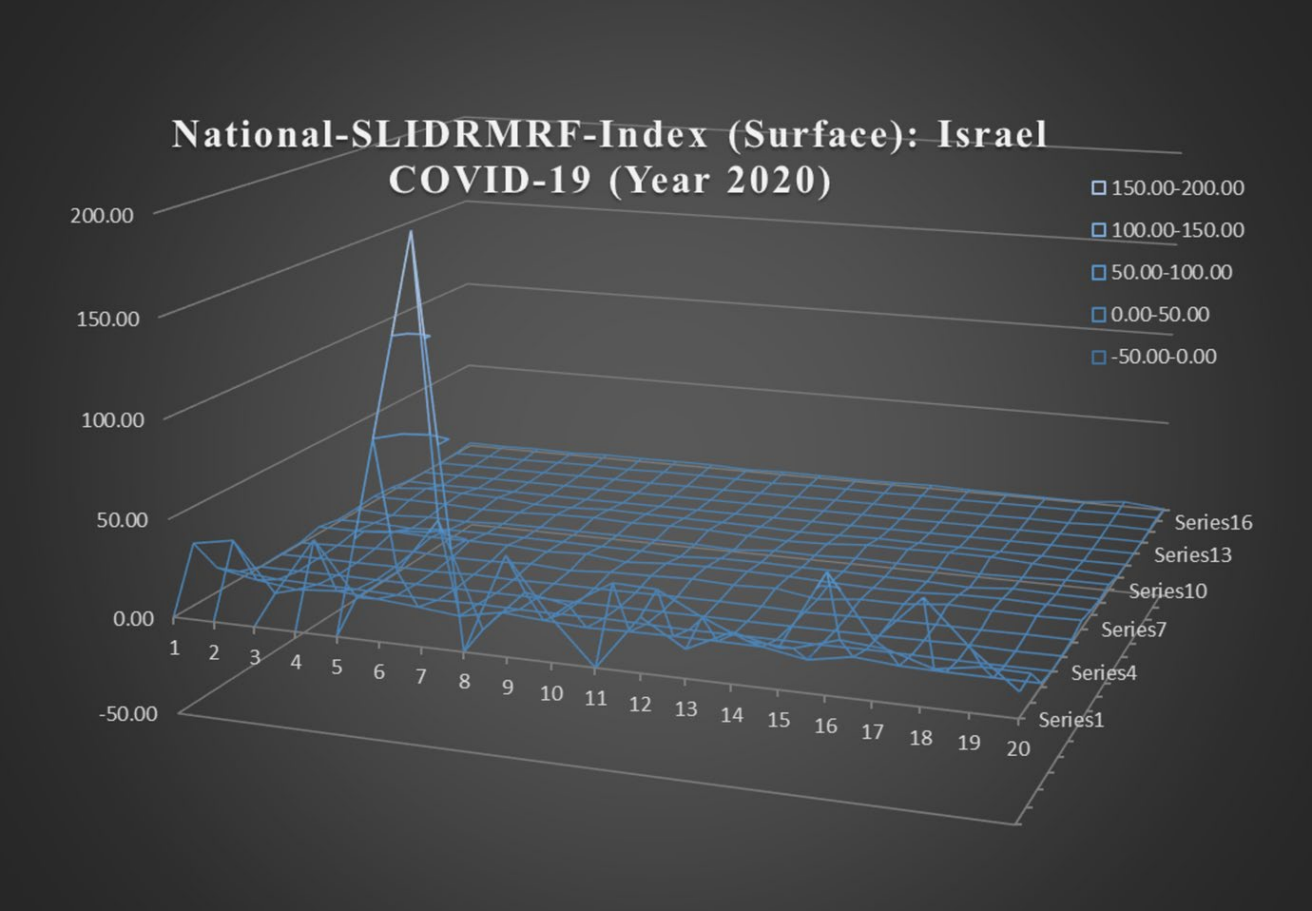
National-SLIDRMRF-Index (Surface): Israel COVID-19 (Year 2020). *Source* Ministry of Health of Israel (2021), and WHO (2021)

However, this period of time in the analysis was the year 2020. The general objective of National-SLIDRMRF-Index is to observe the link between the final impact of religious festival celebrations and the increment of COVID-19 cases in the short run. In applying National-SLIDRMRF-Index to Israel, we focus on the impact of the three major religious festivities celebrations such as Judaism, Christianity, and Islam on the number of COVID-19 cases in Israel. Evaluating the National-SLIDRMRF-Index to Israel, we can observe that in Fig. 5, at the end of the year 2020 there exists an uncontrolled increment of COVID-19, which may be substantial given their urgent measures to implement major controls on the national level by the government in Israel.

**Map. 1:**
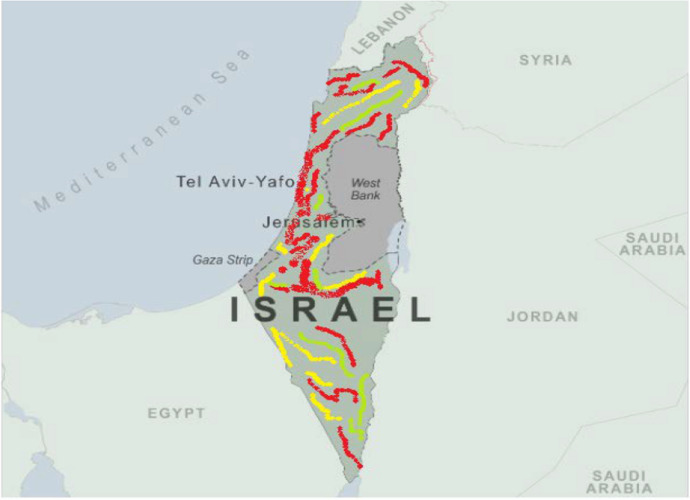
Map of Israel and the spread of COVID-19 in different districts according the number of cases concentration. *Source* Center for Diseases Control and Prevention (2021), Ministry of Health of Israel (2021), and WHO (2021)

The religious festivities celebrations, in turn, can significantly affect the expansion of COVID-19 in different districts of Israel; according to Map 1 we can see red color represents a critical situation, yellow color shows a high possibility in the fast spread of COVID-19 anytime, and green color exhibits a light number of COVID-19 cases. It is possible to observe especially in Jerusalem and large cities of Israel with high population density such as Tel Aviv and Haifa, presented in a red color in Map 1.

The results of the National-SLIDRMRF-Index show that the probability of infection from COVID-19 among the three major religious groups presents the next results: Jewish (0.91), Christians (0.83), and Muslims (0.95) according to preliminary calculations. The case of small cities or far districts in Israel can clearly show less damage from COVID-19, especially in the South part of Israel. However, in the North and middle parts of Israel, the average probability in these two regions of Israel to be infected by COVID-19 is equal to 0.97. The large cities and their population concentration encourage the fast spread of COVID-19, according to our index.

In terms of the National-SLIDRMRF-Index, North of Israel the National-SLIDRMRF-Index (0.71) and the Central part of Israel the National-SLIDRMRF-Index (0.97) were high for Israel in the period 2020–2021. Obviously, the North and central parts of Israel are the two regions most affected by COVID-19. Such a high level of COVID-19 suffered by Israel can be attributed to the high level of population concentration in different religious festivities celebrations informally. Furthermore, Israel showed a high level of COVID-19 in Jerusalem and Tel Aviv during the same period. There are high levels of COVID-19 in Israel against different religious festivities celebrations in public places, but the reunions continue among families and neighbors from the same religion, making it difficult to stop COVID-19 in Israel.

## Conclusion and Recommendations

Our paper concludes that it is possible to evaluate how religious festivities can generate a certain impact in the increment of COVID-19 cases through the National-SLIDRMRF-Index. These new analytical tools can help us to assess the religious festivities celebrations in times of pandemic crisis. At a broader level, the National-SLIDRMRF-Index is useful for mathematically measuring and graphically illustrating the spread of COVID-19 in different religious groups before and after implementing any movement control order (MCO), including its final impact on different religious groups. Finally, our index’s application to Israel that the relaxation on any religious festivity celebration can generate a high level of COVID-19 cases in the short run. Finally, the religious festivities celebrations’ main problem is the informal reunions among relatives, close friends, and neighbors from the same religion, respectively.

However, our recommendations to the Israel government are followed by:

1. Mores restrictions in any religious festival or gathering in different locations of Israel, especially in Jerusalem.
2. Daily mapping of different red areas with high concentration of COVID-19-infected cases to keep all citizens informed about it.
3. A constant monitoring of different religious events.
4. The uses of social distances and mask.
5. Police and Army check points to control the mobility of people traveling in Israel.
6. Measure of temperature before any religious activity starting.
7. The uses of drones to evaluate the temperature of massive agglomerations in any religious festival.
8. The constant monitoring of temperature in the prayers hours.
9. The constant disinfect and cleaning of holy places such as the synagogues, churches, and mosques.
10. The implementation of penalties and fines to people who never follow the vaccination, social distance, and uses of the mask.
11. Centers to test the COVID-19 near to any synagogue, church, and mosque.
12. Major controls in weeding’s or any religious gathering.

Finally, we can say that this paper can contribute to reduce effectively the cases of COVID-19-infected cases under a major control of religious celebrations using strict regulations and fines to keep lower rates of COVID-19-infected cases in the short run in Israel.

## Data Availability

Available upon request.
